# Comprehensive Study on Quantitative Evaluation of Oral Muscle Tissue in Children With Low Tongue Posture Using Cone Beam Computed Tomography: A Comprehensive Study on Nasal Ventilation Conditions Using Computational Fluid Dynamics

**DOI:** 10.1111/joor.70182

**Published:** 2026-03-17

**Authors:** Yukari Suzuki, Takamasa Kitamura, Kei Maeo, Tomohiro Kizuki, Mayu Noda, Hitomi Kuramoto, Yuki Akazawa, Hitomi Ishii, Akihiro Haga, Ryuzo Kanomi, Tomonori Iwasaki

**Affiliations:** ^1^ Department of Pediatric Dentistry, Institute of Biomedical Sciences Tokushima University Graduate School Tokushima Japan; ^2^ Kanomi Orthodontic Office Himeji Japan; ^3^ Department of Medical Imaging Physics, Graduate School of Biomedical Sciences Tokushima University Tokushima Japan

**Keywords:** computational fluid dynamics, cone beam computed tomography, low tongue, nasal obstruction, oral muscle tissue

## Abstract

**Background:**

Low tongue posture (LTP) results from nasal obstruction and impaired oral muscle function. However, the relationship between nasal obstruction‐independent LTP and oral muscle function remains unclear.

**Objective:**

In this study, we aimed to comprehensively examine the relationship between nasal obstruction‐independent LTP and oral muscle function by evaluating LTP, nasal ventilation conditions, and oral muscle function based on the cross‐sectional area of the muscle‐fat tissue surrounding the oral cavity.

**Methods:**

Participants included 28 children (LTP group: mean age: 8.71 years) with LTP and 24 children (control group: mean age: 8.89 years) without LTP. Nasal airway ventilation conditions were evaluated using nasal fluid analysis of the cone beam computed tomography (CBCT) data. Oral muscle function was assessed by measuring the cross‐sectional area of muscle‐fat in the upper and lower lips, upper and lower oral vermilions, and tongue using CBCT data.

**Results:**

Compared with the control group, the LTP group exhibited higher nasal airway pressure and reduced muscle cross‐sectional areas in the lower lip, lower orbicularis oris, and tongue. The LTP group without nasal obstruction had significantly smaller muscle cross‐sectional areas of the lower lip and tongue than the group without LTP. The group without LTP but with nasal obstruction had a significantly larger tongue muscle cross‐sectional area than the LTP group.

**Conclusion:**

LTP can involve nasal obstruction and reduced muscle components in the lower lip, orbicularis oris, and especially the tongue, being related to lower muscle function. The findings could help improve the management of LTP‐associated disorders.

## Graphical abstract

Measurement of nasal airway computed fluid dynamics, muscle‐fat tissue analysis in this study. Four typical examples illustrating the relationship between low tongue posture, nasal ventilation condition, and perioral muscle‐fat tissue are demonstrated in this study. A. Children without nasal ventilation impairment and low tongue posture Upper panel: Nasal fluid analysis shows no high pressure, indicating no nasal ventilation impairment. Lower panel: Muscle‐fat tissue analysis of the lips and tongue shows abundant tongue muscle tissue distribution and functional tongue development. B. Children with low tongue position but no nasal ventilation impairment Upper panel: Nasal fluid analysis shows no high pressure, indicating no nasal ventilation impairment. Lower panel: Shows sparse muscle tissue distribution in the tongue and lower lip, indicating delayed functional development. C. Children with nasal obstruction but no low tongue posture. Upper panel: Nasal fluid analysis shows high nasal pressure, indicating nasal obstruction. Lower panel: Tongue has abundant muscle tissue, indicating functional development. D. Children with nasal airway obstruction and low tongue posture Upper panel: Nasal fluid analysis shows high nasal pressure, indicating nasal airway obstruction. Lower panel: Reduced tongue muscle tissue indicates delayed functional development of the tongue.

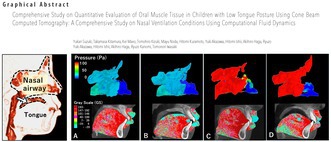

## 1 Introduction

Low tongue posture (LTP) in childhood is observed in mouth breathing children [1, 2, 3]. Its long‐term persistence affects maxillofacial morphology and dental arch form, leading to malocclusion [4, 5]. Recent report [6], have also suggested that it may be a contributing factor to pediatric obstructive sleep apnea (OSA). Therefore, improvement of LTP in children is considered to contribute to healthy growth in maxillofacial and dental morphology, oral function, and respiration.

Regarding LTP causes, maxillary dental arch narrowing has been associated with cases without nasal obstruction [4]. Reports indicate that not only dental arch narrowing, but also nasal obstruction is involved [6].

Furthermore, children exhibiting LTP without maxillary dental arch narrowing or nasal obstruction have been reported. Since tongue position improved with orofacial myofunctional therapy (OMFT), reduced oral muscle function may influence LTP [7].

Children with mouth breathing and LTP have been reported to have weak lip closure force [8] and low tongue pressure [9, 10]. However, the diagnosis of mouth breathing is based on interviews and visual inspection, without objective assessment of nasal airway patency. Consequently, the respective relationships between LTP and nasal obstruction, and between LTP and oral muscle function remain unclear.

Therefore, clarifying the relationship between LTP and oral muscle function facilitates the identification of LTP children requiring OMFT, helps pinpoint the oral muscles needing intervention, and leads to effective improvement of LTP through OMFT.

Therefore, in this study, to clarify the relationship between LTP and oral muscle function in children with LTP who did not exhibit maxillary dental arch narrowing or nasal obstruction—factors traditionally considered causes of LTP—we adopted a novel method for oral muscle function analysis using cone beam computed tomography (CBCT) image data to measure the areas of muscle and fat tissue in the lips and tongue.

## 2 Methods

This study was approved by the Tokushima University Hospital Medical Research Ethics Review Committee (Approval No. 4393–5). Because this study was retrospective, the requirement for informed consent was waived. The participants were 460 children aged 7–12 who underwent CBCT examinations at a collaborative orthodontic clinic for orthodontic treatment evaluation between January 2007 and March 2013. Children with LTP were selected as the study group, and children without LTP were selected as the control group.

### 2.1 Inclusion Criteria

Inclusion criteria were as follows:

1. Presence of LTP: Regarding the definition of low tongue posture, studies report an intraoral airway volume (IAv) ≥ 1 cm^3^ in children with nasal airway obstruction [11]. However, another report [6]. Found volumes < 1 cm^3^ in cases without malocclusion or in cases with improved nasal airway obstruction. Previous research investigating the relationship between nasal airway patency and LTP [7], demonstrated high sensitivity and specificity when defining LTP as ≥ 1 cm^3^. Therefore, in this study also, we adopted ≥ 1 cm^3^ as the definition for low tongue posture.
2. No history of orthodontic treatment.
3. No severe dental malocclusion or skeletal malocclusion.
4. No systemic diseases affecting the craniofacial region.

Exclusion criteria were as follows:

1. Presence of adenoid hypertrophy [12, 13, 14], or palatine tonsil hypertrophy [14, 15].
2. History of adenoidectomy or tonsillectomy.
3. Presence of systemic disease
4. Confirmation of swallowing, tongue position, or other body movements on CBCT images.
5. Cases showing maxillary sinus hypertrophy.

As a result, 28 children showed LTP (LTP group: 8.71 ± 1.09 years old, 11 boys), while 24 children did not (control group: 8.89 ± 1.13 years old, 11 boys).

### 2.2 CBCT Scan

CBCT imaging was performed only in cases where orthodontic treatment was deemed necessary to minimise radiation exposure and examine maxillofacial morphology, nasal airway and pharynx, condition of the paranasal sinuses, and dental problems.

CBCT scans were performed with participants seated, ensuring that the Frankfort horizontal plane was parallel to the floor [16], with a craniovertebral angle between 95°and 105° [17]. Participants were instructed to maintain a resting tongue position during imaging. Images affected by swallowing or body movement, which produced obvious artefacts, were excluded; only consistent images were included for analysis across all participants in this study. CBCT scans were acquired using a CBCT unit (Asahi Roentgen Japan Alphard 3030) at a maximum voltage of 80 kV, a maximum of 2 mA, an exposure time of 17 s, and a voxel dimension of 0.39 mm. Data were sent directly to a personal computer and stored in the Digital Imaging and Communications in Medicine (DICOM) format.

### 2.3 Morphological Evaluation

A three‐dimensional (3D) coordinate system and 3D images were constructed with a medical image analysis system (ImagnosisVE, Kobe, Japan) using CBCT data [16]. From these constructed cephalometric images, the anteroposterior positions of the maxilla and mandible were evaluated using the SNA angle (Point A: deepest midline point of the premaxilla between the anterior nasal spine and prosthion), SNB angle (Sella‐Nasion to B Point Angle), ANB angle (A point to B Point Angle), and FMA (Frankfort mandibular plane angle).

Furthermore, a 3D model of the maxillofacial region was constructed from the CBCT data using volume rendering software (INTAGE Volume Editor Cybernet, Tokyo, Japan). The width of the maxillary dental arch width (intramaxillary molar width) and IAv, which represent the airway space between the tongue and palate, were measured using CBCT [7].

**FIGURE 1:**
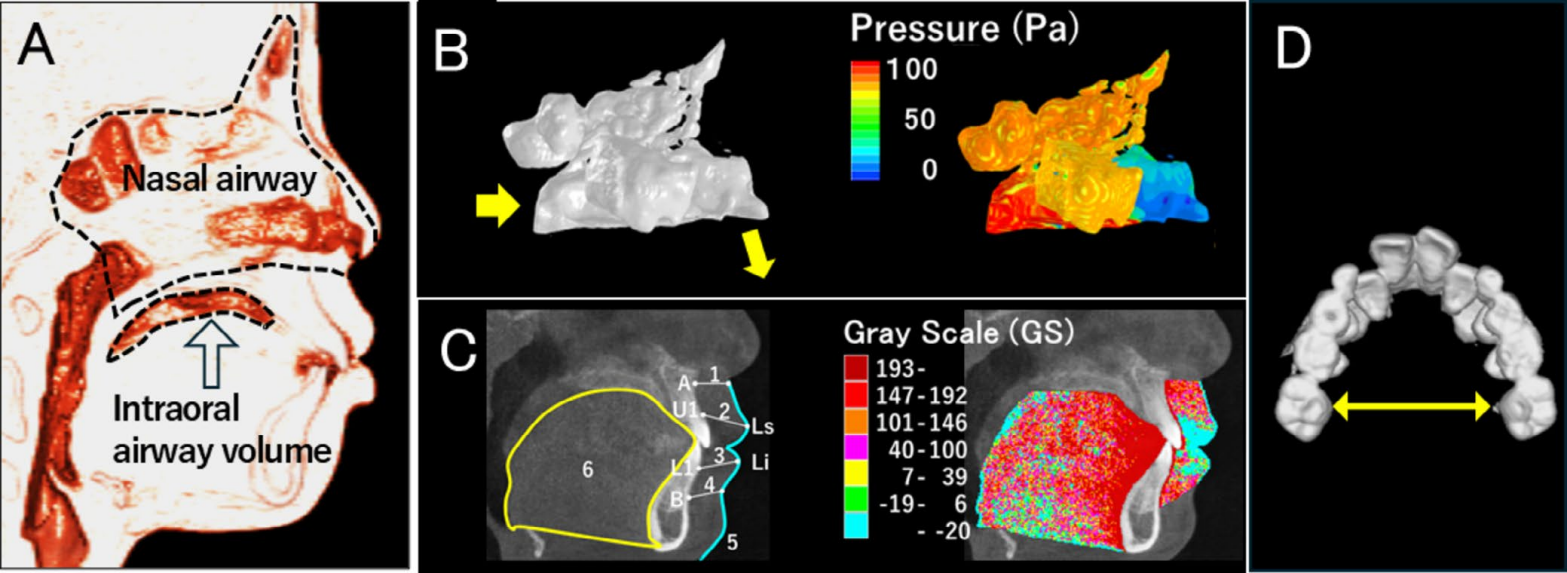
Measurement of nasal airway computed fluid dynamics, muscle‐fat tissue analysis, and maxillary dental arch width performed in this study. (A) Constructing 3D images from CBCT data and extracting models of the nasal airway, intraoral airway, and maxillary dental arch width. (B) Computed fluid dynamics in the nasal airway using the 3D nasal airway model, displaying pressure values to evaluate nasal ventilation condition. (C) Image mapping of muscle‐fat tissue corresponding to the upper and lower lips, the upper and lower orbicularis oris, and tongue in the sagittal midface from CBCT data. Line 1: Straight line from Point A to the base of the nose. Line 2: Straight line from the cervical region of the maxillary central incisor (U1) to the most protruding point of the upper lip (Ls). Line 3: Straight line from the cervical region of the mandibular central incisor (L1) to the most protruding point of the lower lip (Li). Line 4: Straight line from point B to the mental‐labial groove. Line 5; Soft tissue contour (light blue solid line). Line 6; Tongue contour (yellow solid line). Upper orbicularis oris; Between line 1 and line 2. Upper lip; Between line 2 and the lower edge of the upper lip, Lower lip; Between line 3 and the upper edge of the lower lip. Lower upper orbicularis oris; Between line 3 and line 4. (D) Measurement of maxillary dental arch width: Inter first‐molar distance.

The 3D nasal airway model was converted into a smoothed model using mesh morphing software (DEP Mesh Works/Morpher; IDAJ, Kobe, Japan), maintaining patient‐specific airway shape patterns, with approximately 800 000 triangular and quadrilateral mesh elements (Figure 1).

The nasal cavity 3D model was exported in stereolithography format and used in computational fluid dynamics (CFD) to analyse nasal ventilation using fluid dynamics software (Phoenics; CHAM Japan, Tokyo, Japan) [7].

In this simulation, air flows horizontally into the posterior nares and is exhaled through both nostrils. The flow was assumed to be Newtonian, homogeneous, and incompressible. The elliptic staggered equation and continuity equation were employed. CFD analysis of the nasal airway using PHOENICS was performed under the following conditions: (1) For the flow rate, previous studies [18, 19] demonstrated that in children with nasal breathing difficulties, the upper airway resistance flow pressure exceeds 100 Pa (1 Pa = 0.01 cmH2O) at an inflow of 200 mL/s (resistance of 5.0 cmH2O/L/s). Therefore, 200 mL/s was used; (2) walls were non‐slip, and (3) simulations were repeated 300 times to calculate the average value. Convergence was monitored by normalising the magnitude of the mass and momentum handling residual sources by their respective inflow fluxes. Iterations were continued until the total residual was less than 0.2%.

### 2.4 Definition of Nasal Obstruction

Previous studies [11, 19] have reported the nasal airway resistance of 0.5 Pa/mL/s in elementary school children. Therefore, in our flow rate setting (200 mL/s), nasal obstruction was considered present when the flow rate exceeded 100 Pa [19]. Furthermore, when the continuity of the bilateral nasal airway openings in the 3D nasal airway model was interrupted, it was considered complete obstruction (3D obstruction) [19].

### 2.5 Muscle Tissue Evaluation

**FIGURE 2:**
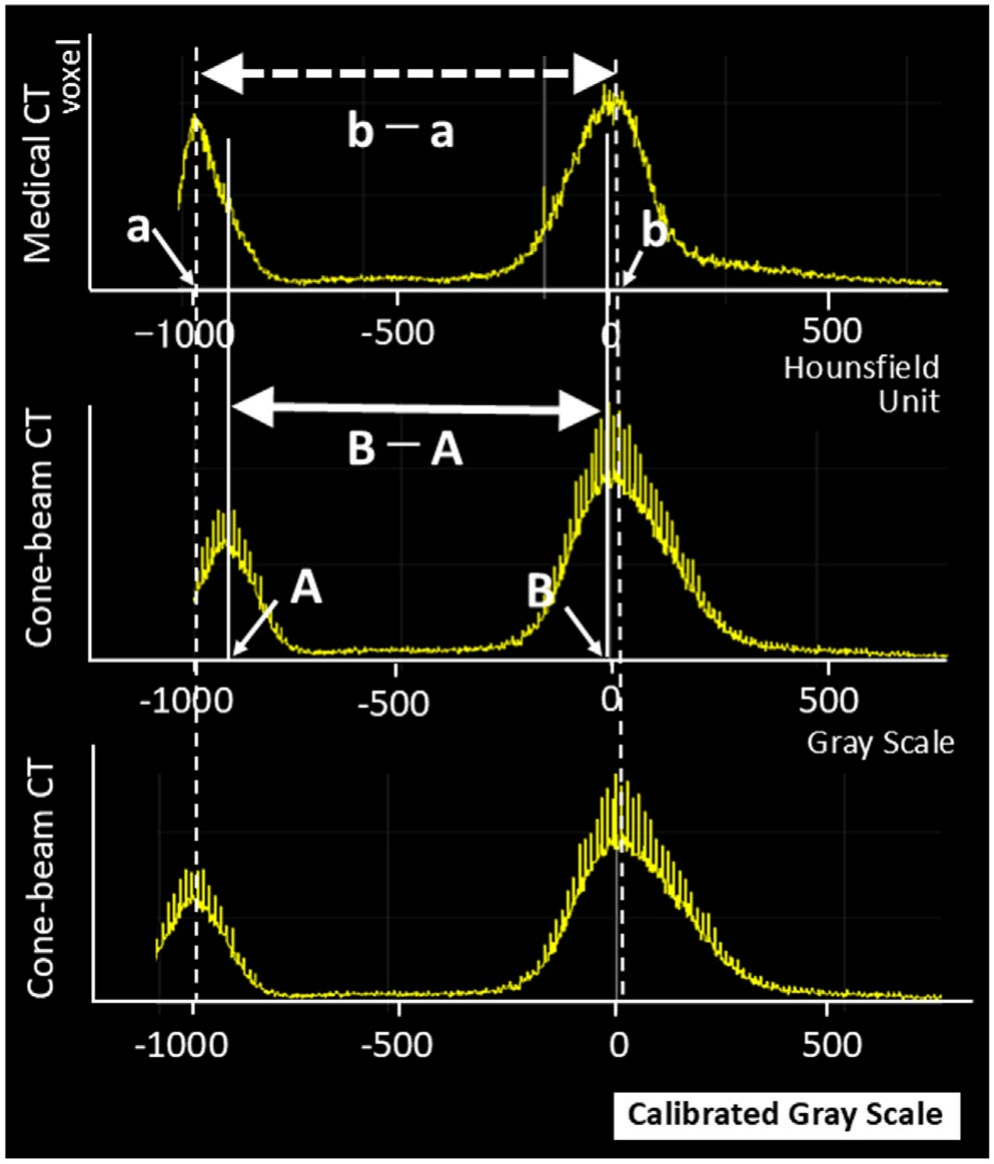
Calculation of cone‐beam computed tomography (CBCT) grey scale (GS) values corresponding to the medical CT (MDCT) Hounsfield unit (HU) Values. Top: Histogram peaks for the air and facial regions in MDCT (Table A1). Histogram peak corresponding to the air regions in MDCT (−999.4 ± 4.4 HU), b; Histogram peak for facial soft tissue in MDCT (36.3 ± 2.8 HU), b‐a; Distance between peaks for the air and facial soft tissue regions (−1035.7 ± 5.1 HU). Middle: Histogram peaks for air and facial soft tissue regions in CBCT (Appendix 1). A; Histogram peak corresponding to the air region in CBCT (−927.9 ± 4.0 GS), B; Histogram peak of the facial soft tissue region in CBCT (36.8 ± 5.1 GS), B‐A; Distance between the peaks of the air region and facial soft tissue region (−964.7 ± 6.7 GS). Lower row: CBCT GS values calibrated to correspond to the MDCT HU values. The distance between the CBCT histogram peaks was adjusted to match the distance between the air region histogram peak and the facial soft tissue histogram peak in MDCT. Using this unified scale, CBCT GS values corresponding to MDCT HU values indicating muscle and fat were calculated.

In this study, image analysis software SliceOmatic (Tomovision, Magog, Canada) was used to calculate the muscle‐fat tissue area of the upper lip, lower lip, orbicularis oris muscle, and tongue from CBCT grayscale values [20]. Previous reports on the relationship between CBCT grayscale values and medical CT (MDCT) HU values have shown strong correlations between the two values [21]; however, it has also been reported that GS values do not correspond to HU values [21]. Therefore, to improve image consistency between CBCT and MDCT, this study standardised the acquisition conditions [22], including using the same device, subjects of similar age, and regions of interest (ROI) of comparable size. Peak HU and GS values for the air and facial regions were measured from each device. As a result, adjusted grayscale values for CBCT were calculated that corresponded well to the MDCT HU values indicating muscle and fat (Figure 2, Supplementary Appendix Table A1). Therefore, the GS values assigned to each muscle‐fat tissue in this study were judged to be sufficiently reliable as values corresponding to the MDCT HU values. The GS values for each tissue and their corresponding HU values are listed in the Supplementary Appendix (Table A2).

### 2.6 Statistical Analysis

For comparisons between two groups, *t*‐tests and Mann–Whitney U tests were performed. Fisher's exact test was used to determine the incidence of nasal obstruction and LTP. *p* < 0.05 was considered statistically significant for all tests.

To ensure reliability, the same evaluator (Y.S.) repeated all the measurements. After repeating the measurements, another author (T.I.) verified the accuracy. If additional measurements were necessary, the same evaluator (Y.S.) repeated the measurements, and the measurement error was calculated using the Dahlberg formula [23].

### 2.7 Measurement Error

Measurement error in intra‐individual (inter‐individual) measurement items in this study showed that the maxillary dental arch width, IAv, nasal airway pressure, and muscle‐fat tissue area were 0.055 mm (0.072 mm), 0.045 mm^2^ (0.055 mm^2^), 1.823 Pa (2.342 Pa), and 0.423 mm^2^ (0.723 mm^2^), respectively. According to all repeated analyses, the method error was considered negligible.

Power analysis was performed using G*Power software (version 3.1.9.7; Franz Faul, Universitat Kiel, Germany), calculating the error (1‐β error = 0.80; α = 0.05; two‐tailed *t*‐test). The appropriate sample size was 18 participants.

## 3 Results

### 3.1 Comparison Between the LTP Group and the Control Group

**FIGURE 3:**
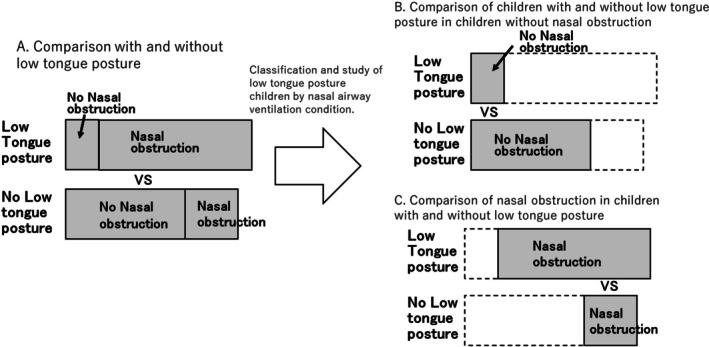
(A) Comparison based on the presence or absence of low tongue posture. Children with low tongue posture include those with nasal obstruction and those without nasal obstruction. Children without low tongue posture also include those with nasal obstruction and those without nasal obstruction. (B) Comparison of children with low‐positioned tongue and children without low‐positioned tongue in children without nasal obstruction, after classification by nasal airflow status in addition to the presence/absence of low‐positioned tongue. (C) Comparison of children with low‐positioned tongue and children without low‐positioned tongue in children with nasal obstruction, after classification by nasal airflow status in addition to the presence/absence of low‐positioned tongue.

IAv was significantly larger in the LTP group (3.94 ± 1.91 cm^3^) than in the control group (0.02 ± 0.06 cm^3^) (Table 1, Figure 3A). Nasal airway pressure was significantly higher in the LTP group than in the control group.

**TABLE 1:** Comparison between two groups without nasal obstruction.

		Comparison of all children with and without low tongue posture					Comparison of without nasal obstruction in children with and without low tongue posture					Comparison of nasal obstruction in children with and without low tongue posture				
		Without low tongue posture: Control group		Low tongue posture: LTP group		*t*‐test or Mann–Whitney U test	Without low tongue posture without nasal obstruction		Low tongue posture without nasal obstruction		*t*‐test or Mann–Whitney U test	Without low tongue posture with nasal obstruction		Low tongue posture with nasal obstruction		*t*‐test or Mann–Whitney U test
		*n* = 24		*n* = 28			*n* = 17		*n* = 5			*n* = 7		*n* = 23		
		Mean	SD	Mean	SD	*P*	Mean	SD	Mean	SD	*P*	Mean	SD	Mean	SD	*P*
Maxillary dental arch width (mm)		34.07	2.03	34.09	2.13		33.56	2	32.3	1.7		35.3	1.62	34.48	2.04	
Intraoral airway volume (cm^3^)		0.02	0.06	3.94	1.91	< 0.001	0.02	0.05	4.05	1.62	0.005	0.03	0.09	3.91	2	< 0.001
Nasal airway Pressure (Pa)		95.57	163.53^a^	395.25	311.97^b^	< 0.001	33.59	14.67	53.3	34.56		271.17	258.67^a^	526.77	264.61^b^	
Each tissue area (mm^2^)																
Tongue	Total	1993	156.6	1730.6	146	< 0.001	2025.7	147.2	1834.9	148	0.019	1913.5	160.6	1707.9	138.4	0.003
	Low density fat	140.8	62.1	158	78.7		140.3	59.1	210.2	128.5		142.1	74.1	146.6	62.1	
	Normal density fat	97.4	27.6	93.8	36.1		99.1	28.8	74	33.8		93.3	26.1	98.1	35.8	
	Very low density muscle	181.2	34.7	165.9	51.6		186.7	35	144.2	55.7	0.05	167.7	32.5	170.6	50.8	
	Low density muscle	449.8	59.8	390.5	71.2	0.002	464.5	58.5	364.2	74.4	0.005	413.9	49.7	396.3	70.8	
	Normal density muscle	344.6	34.3	287.5	30.1	< 0.001	352.8	34.1	300.9	35.5	0.008	324.8	27.9	284.6	28.8	0.003
	High density muscle	268.8	26.5	219.9	30.5	< 0.001	272.5	25.3	248.1	13.8		259.7	29.1	213.8	29.8	0.001
	Very high density muscle	510.3	56.3	415	95.8	< 0.001	509.7	59.1	493.3	61.4		511.8	53.1	397.9	94.3	0.005
Lower lip	Total	92.8	18.7	93.3	17.7		90.7	20.2	82.6	7		98	14.3	95.6	18.6	
	Low density fat	35.2	10.8	43.7	15.6	0.027	32.4	8.9	43.8	10.1	0.024	42.1	12.5	43.6	16.8	
	Normal density fat	9.1	2.9	8	3.3		8.1	2.5	8.5	2.9		11.4	2.8	7.9	3.4	0.019
	Very low density muscle	11.4	3.2	9.8	3.4		10.9	3.1	9.3	2.3		12.5	3.3	9.9	3.6	
	Low density muscle	16.5	6.9	14.1	6.6		16.6	6.9	9.8	4.7		16.1	7.5	15	6.7	
	Normal density muscle	7	3.9	6.3	3.9		7.3	4	3.4	2.4		6.2	3.7	7	3.9	
	High density muscle	3.7	2.2	3.4	2.2		4.1	2.3	2	1		2.9	1.8	3.7	2.3	
	Very high density muscle	10	4.7	8	4.4		11.2	4.5	5.8	2.7	0.019	7	3.8	8.5	4.5	
	Total	72.5	16	69	17.8		73.8	15.6	62.9	11.4		69.5	17.9	70.3	18.9	
Lower rbicularis oriscularis oris
	Low density fat	9.9	5.6	13.6	9.7		9.3	5.1	11.5	2.9		11.3	6.9	14.1	10.6	
	Normal density fat	4.2	2.2	4.4	2.1		4.1	2.3	4.6	3.1		4.4	1.9	4.3	2	
	Very low density muscle	6.6	2.4	7	2.3		6.7	2.6	7.5	2.3		6.3	1.8	6.9	2.3	
	Low density muscle	14.4	4.5	14.8	4.6		14.5	4.5	13.4	2.2		14.2	4.8	15.1	4.9	
	Normal density muscle	11.4	3.9	10.5	4.2		11.8	4.3	9.1	4.2		10.4	3	10.8	4.2	
	High density muscle	9.9	4.1	7.6	4.1	0.042	10.5	4.4	6.3	3.1		8.5	2.7	7.8	4.3	
Very high density muscle	16.1	8.6	11.1	6.2	0.02	16.8	9.8	10.5	5.5		14.5	5.3	11.3	6.5	

*Note:* All these tissue areas of upper lip and upper orbicularis oris were not significant differences each between the groups.

^a^ one case 3D nasal obstruction,

^b^ 10 cases 3D nasal obstruction.

In histological evaluation, comparison between the LTP and control groups showed that the total cross‐sectional areas of the tongue were 1730.6 ± 146.0 mm^2^ in the LTP group and 1993.0 ± 156.6 mm^2^ in the control group, with the LTP group being significantly smaller (Table 1, Figure 3A). Comparing individual components, the tongue showed significantly less muscle tissue in the LTP group than the control group for all muscles (low‐density muscle, normal‐density muscle, high‐density muscle, and very high‐density muscle), and the total tongue area was also smaller.

In the areas corresponding to the upper lip and the upper orbicularis oris, no significant difference in the total cross‐sectional area was observed between the groups. However, in the lower orbicularis oris, the cross‐sectional area of the muscle (high‐density and very high‐density muscles) was significantly smaller in the LTP group than in the control group.

### 3.2 Relationship Between LTP and the Presence of Nasal Obstruction

Regarding the incidence of nasal obstruction in relation to the presence of LTP, Fisher's exact test revealed that 23 children in the LTP group had nasal obstruction (10 with complete obstruction, 13 with 100 Pa or greater), while five had no nasal obstruction. The incidence of nasal obstruction in the children with LTP was 82.1% (23/28). In the control group without LTP, seven children had nasal obstruction (one with complete obstruction, seven with resistance ≥ 100 Pa), while 17 children had no nasal obstruction, yielding a nasal obstruction incidence of 29.1% (7/24). The LTP group had a significantly higher incidence of nasal obstruction (Table 2).

**TABLE 2:** Incidense of nasal obstruction between two groups.

		Without low tongue posture: Control group	Low tongue posture: LTP group	Fisher’s exact test
		*n* = 24	*n* = 28	*P*
Nasal airway (*n*)	Obstruction	7	23	< 0.001
	No obstruction	17	5	
Nasal obstruction incidence (%)		29.1% (7/24)	82.1% (23/28)	

*Note:* 100 Pa or higher and 3D obstruction were defined as nasal obstruction.

### 3.3 Comparison of LTP Presence in Children Without Nasal Obstruction

**FIGURE 4:**
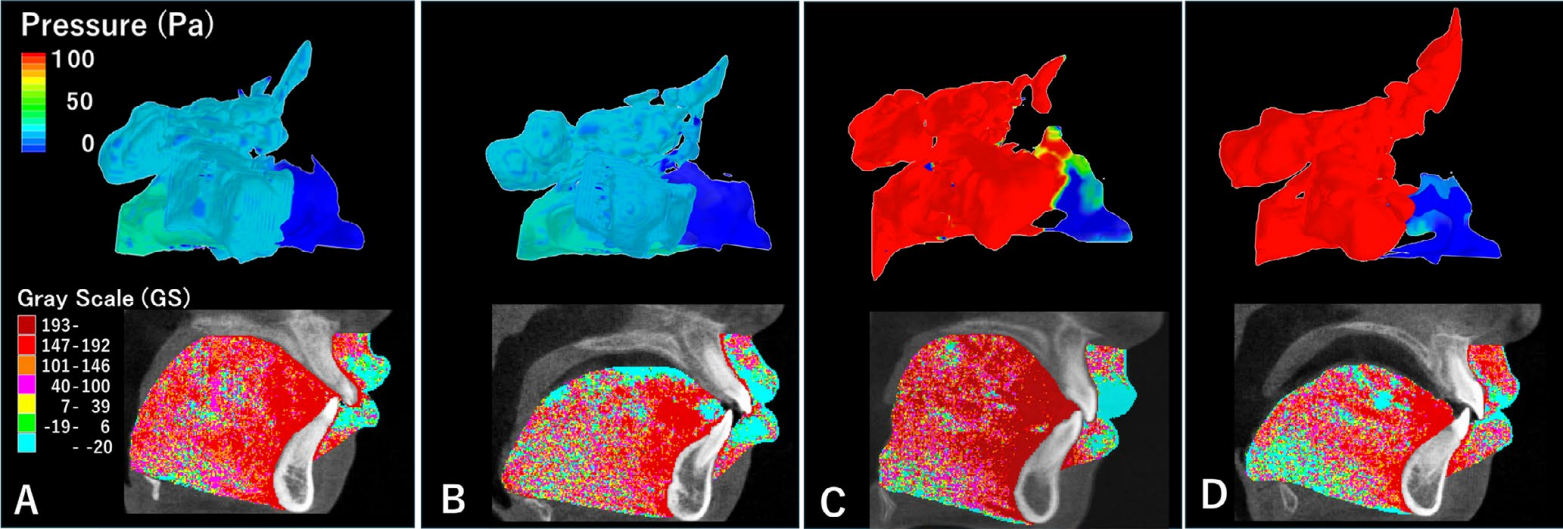
Four typical examples illustrating the relationship between low tongue posture, nasal ventilation status, and perioral muscle‐fat tissue demonstrated in this study. (A) Children without nasal ventilation impairment and no low tongue posture: Upper panel: Nasal fluid analysis shows no high pressure, indicating no nasal ventilation impairment. Lower panel: Muscle‐fat tissue analysis of the lips and tongue. Shows abundant tongue muscle tissue distribution and functional tongue development. (B) Children with low tongue posture but no nasal ventilation impairment. Upper panel: Nasal fluid analysis shows no high pressure, indicating no nasal ventilation impairment. Lower panel: Shows sparse muscle tissue distribution in the tongue and lower lip, indicating delayed functional development. (C) Children with nasal obstruction but no low tongue posture. Top: Nasal fluid analysis shows high nasal pressure, indicating nasal obstruction. Bottom: Tongue has abundant muscle tissue, indicating functional development. (D) Children with nasal airway obstruction and low tongue posture. Upper panel: Nasal fluid analysis shows high nasal pressure, indicating nasal airway obstruction. Lower panel: Reduced tongue muscle tissue indicates delayed functional development of the tongue.

Children with LTP but without nasal obstruction had a smaller total tongue area and a significantly smaller muscle cross‐sectional area (very low‐density muscle, low‐density muscle, and normal‐density muscle) than children without LTP (Table 1, Figure 3B, Figures 4A and 4B); furthermore, their lower lip had more fat and a significantly smaller muscle cross‐sectional area (very high‐density muscle).

### 3.4 Comparison of Children With Nasal Obstruction Based on Presence/Absence of LTP

A comparison of children with nasal obstruction based on the presence/absence of LTP (Table 1, Figure 3C, Figures 4C and 4D) showed that children with nasal obstruction but without LTP had a significantly larger tongue muscle cross‐sectional area (normal‐density muscle, high‐density muscle, and very high‐density muscle) and a significantly larger total tongue area than children with nasal obstruction and LTP.

## 4 Discussion

Factors influencing LTP include dental arch narrowness [4] and nasal obstruction [6]. Although recent reports suggest that oral muscle function may influence LTP [7], evaluating the nasal airway ventilation condition—which affects LTP—has been challenging. Consequently, it is difficult to investigate the impact of oral function alone on LTP while controlling for this influence. Therefore, to clarify the effect of oral muscle function on LTP, the present study evaluated the dental arch morphology and nasal airway ventilation conditions using fluid dynamics analysis, both of which have been reported to influence LTP. Herein, we conducted a comprehensive investigation by first clarifying the presence or absence of nasal obstruction and then used our newly proposed method to evaluate oral muscle function based on the cross‐sectional area of muscl‐fat tissue (Figures 3B and 3C).

The present study showed that children with LTP exhibited nasal airway obstruction and had a smaller total cross‐sectional area of the tongue, and from the perspective of muscl‐fat tissue, a lower proportion of muscle components in the tongue than children without LTP (Table 1).

However, these results did not differentiate between the respective contributions of nasal obstruction and reduced tongue muscle function as causes of LTP, thus preventing a clear determination of the impact of oral muscle function on LTP (Figure 3A).

Therefore, cases were classified based on the presence or absence of nasal obstruction, and the oral function affecting LTP was evaluated for each nasal airway ventilation condition (Figure 3 bc). The results showed that despite having no nasal obstruction, children with LTP had reduced tongue muscle mass and a smaller total tongue cross‐sectional area. Similar trends were observed in the lower lips. This observation indicates that reduced muscle function in the tongue and lower lip predisposes patients to LTP, even in the absence of nasal obstruction (Table 1, Figure 3B). The present study examined the presence or absence of LTP in children with nasal obstruction (Figure 3C).

The results showed that children with nasal obstruction but without LTP had a higher tongue muscle component and a larger total cross‐sectional area of the tongue. This observation suggests that children with functional tongue development are less likely to develop LTP (Table 1; Figure 3).

Considering these nasal ventilation conditions, we examined the effects of oral muscle function on LTP. These results support that narrowing of the maxillary dental arch [4, 5] and nasal obstruction [6, 7], and delayed functional development of the tongue and lower lip contribute to LTP.

Previous studies on oral muscle function in children with mouth breathing, which is closely related to LTP, have reported reduced tongue pressure [9] and lip closure [8]. Similar findings have been reported in adolescents (16.6 years old) [10]. However, in these reports [8, 9, 10], the diagnosis of mouth breathing associated with LTP was based on visual inspection and questioning. This likely included children with nasal obstruction who breathe through their mouths, habitual mouth breathers without nasal obstruction, and dental mouth breathers who had difficulty closing their lips because of the dental arch morphology [19]. Therefore, the decline in oral muscle function, as indicated by muscle strength, such as tongue pressure and lip pressure in mouth breathing children, shown in these studies, could not clearly establish the relationship between LTP and oral function because the nasal airway ventilation condition was not evaluated. We considered it necessary to assess the nasal airway ventilation conditions.

In the present study, we were able to eliminate the influence of nasal obstruction on LTP and examine solely the influence of oral function by using nasal fluid analysis to evaluate the presence or absence of nasal obstruction.

Children with significant skeletal or dental malocclusion were excluded from the analysis, eliminating those with dental‐related mouth breathing caused by the dental arch morphology. Thus, the children in the present study who showed LTP without nasal obstruction were highly likely to be habitual mouth breathers.

The reduced tongue muscle function identified as a factor in LTP in this study [7] showed a higher improvement rate when combined with OMFT than when RME (rapid maxillary expansion) alone was performed, even in children whose nasal ventilation improved with RME and dental arch expansion. This phenomenon indicates that improving LTP requires addressing dental arch morphology and nasal obstruction, as well as muscle function training, supporting the findings of this study.

### 4.1 Reproducibility of Tongue Posture

There are limitations in accurately determining whether the tongue posture during imaging corresponds to its resting position. However, cases exhibiting typical tongue dorsum shapes were selected based on other factors such as the shape of the tongue dorsum and soft palate, shape of the lips and jaw, and image blurring. Therefore, the determination of resting tongue posture in this study was considered sufficiently reliable.

### 4.2 Clinical Implications

The results suggest an association between LTP and oral muscle dysfunction. Therefore, LTP improvement warrants confirming dental arch morphology and the presence or absence of nasal obstruction, implementing necessary interventions, and conducting oral muscle function training. Notably, children with LTP demonstrated a significant reduction in tongue muscle tissue, suggesting the importance of tongue training in oral muscle function therapy.

Furthermore, as this study focused on LTP, it strongly suggests reduced tongue muscle function. However, this analytical method can evaluate muscle function in other oral regions. Therefore, it can detect functional deficits in areas beyond LTP, such as chewing, swallowing, and articulation. It enables the pinpoint detection of dysfunctional areas, allowing efficient targeted OMFT intervention. As a novel evaluation method, it holds significant potential for assessing muscle function in areas inaccessible to conventional instrument‐based evaluation (submandibular region, mandibular‐lingual muscle groups, soft palate), as well as in elderly patients and disabled individuals (including children) with tracheostomies, where conventional testing is challenging.

### 4.3 Limitations

In this study, there were 5 cases of children without nasal obstruction who showed LTP and were suggested to be associated with impaired oral muscle function, and 7 cases of children with nasal obstruction but well‐developed oral muscle function who did not show LTP. The number of cases was small in each group. Nevertheless, the results demonstrated similar trends regarding the association between oral muscle function development and LTP under the distinct conditions of nasal obstruction and absence of nasal obstruction. Despite the small sample size, the association between oral muscle function and LTP was considered reliable.

Although the pressure values in the CFD analysis demonstrated significant variation, the variation in the pressure values around 100 Pa—the threshold used to determine nasal obstruction—was small. Therefore, this variation presumably did not significantly impact the research results.

Future research should include larger sample sizes and comparative analyses between conventional functional assessments, including lip closure force and tongue pressure, and the muscle function evaluation approach demonstrated in this study.

Furthermore, because this study employed a retrospective design, otolaryngologists could not directly evaluate the ventilation status of the paediatric patients. To validate these findings, prospective studies incorporating otolaryngological evaluation are warranted.

## Author Contributions

Yukari Suzuki: conceptualization, methodology, investigation, data analysis, writing original draft, writing review and editing, project administration. Takamasa Kitamura: methodology, data analysis. Kei Maeo: methodology, investigation, data analysis. Tomohiro Kizuki: methodology, data analysis. Mayu Noda: investigation, data analysis. Hitomi Kuramoto: methodology, data analysis. Yuki Akazawa: methodology, investigation, data analysis. Hitomi Ishii: methodology, data analysis. Ryuzo Kanomi: conceptualization, methodology, resources, supervision. Akihiro Haga: methodology. Tomonori Iwasaki: methodology, supervision, writing review and editing, final approval of the manuscript.

## Funding

This work was supported by Japan Society for the Promotion of Science, JP23K09416, JP25K13316.

## Conflicts of Interest

The authors declare no conflicts of interest.

## Supporting information


**Table A1** CBCT and MDCT air and facial soft tissue area peak values.

**Table A2** Grey Scale and Hounsfield Unit Values for Each Muscle and Fat Tissue.

## Data Availability

The data that support the findings of this study are available from the corresponding author upon reasonable request.
